# An Easy Method of Synthesis Co_x_O_y_@C Composite with Enhanced Microwave Absorption Performance

**DOI:** 10.3390/nano10050902

**Published:** 2020-05-08

**Authors:** Wenli Bao, Cong Chen, Zhenjun Si

**Affiliations:** 1 School of Materials Science and Engineering, Changchun University of Science and Technology, No. 7989, Weixing Road, Changchun 130022, China; cccmcx@163.com; 2 School of Physics and Electronic Information Engineering, Qinghai Nationalities University, Xining 810007, China

**Keywords:** controllable interfaces, magnetic Co_x_O_y_@C composite, width band EM absorption, interfacial polarization, tunable content

## Abstract

Design of interface-controllable magnetic composite towards the wideband microwave absorber is greatly significance, however, it still remains challenging. Herein, we designed a spherical-like hybrids, using the Co_3_O_4_ and amorphous carbon as the core and shell, respectively. Then, the existed Co_3_O_4_ core could be totally reduced by the carbon shell, thus in Co_x_O_y_ core (composed by Co and Co_3_O_4_). Of particular note, the ratios of Co and Co_3_O_4_ can be linearly tuned, suggesting the controlled interfaces, which greatly influences the interface loss behavior and electromagnetic absorption performance. The results revealed that the minimum reflection loss value (RL_min_) of −39.4 dB could be achieved for the optimal Co_x_O_y_@C sample under a thin thickness of 1.4 mm. More importantly, the frequency region with *RL* < −10 dB was estimated to be 4.3 GHz, ranging from 13.7 to 18.0 GHz. The superior wideband microwave absorption performance was primarily attributed to the multiple interfacial polarization and matched impedance matching ability.

## 1. Introduction

With the rapid development of electronic devices, e.g., radar communications, wireless and local area network, etc., the electromagnetic (EM) pollutions are becoming serious, especially for regarding as “health killer” and for simultaneously disturbing normal operation of other precious devices [1,2,3]. In case to reduce electromagnetic pollution, more attempts have been paid on exploration of functional materials with the strong dielectric or magnetic loss ability, so that the EM waves can be converted into heats by these materials [4,5]. Commonly, the efficiency of conversion from EM to heats is termed as reflection loss (*RL*) value. As an ideal EM material, it is commonly requested to a width band absorption (frequency region with *RL* < −10 dB, noted that −10 dB is regarded as the commercial standard with a conversion efficiency of 90%) [6,7]. Meanwhile, a thin thickness is quite vital to reduce the weight of absorption layer [8]. Nowadays, it is widely believed that EM performance is primarily influenced by both component and nanostructure; thus the strategy of component/nanostructure has been a general way to develop EM absorber [9]. As a desirable candidate, magnetic hybrids have been widely investigated, because of their dual magnetic and dielectric loss ability [10,11]. E.g., Jia et al. employed an in situ growth route to prepare Fe/ZnFe_2_O_4_ hybrids, and the frequency region (*f_s_*) was up to 6.2 GHz under a matched thickness of 1.5 mm [12]. Zhao and coworker utilized a facile polyol reduction approach to prepare Co/CoO composite, and reported that *f_s_* value was 4.2 GHz under a thickness of 1.7 mm [13]. Li et al. combined magnetic FeCo with SiO_2_ and polypyrrole; the measured *f_s_* was ~6.8 GHz with a thickness of 2.5 mm [14]. For these desirable *f_s_* values, the synergistic effect between magnetic and dielectric loss played a key role on the EM attenuation ability. Besides, the improved complex permeability value (*μ_r_*) was also benefited to the impedance matching behavior [15]. In this case, more EM waves could enter into the interior of EM absorption layer for the subsequent attenuation. For this purpose, magnetic components are always decorated with a series of high dielectric material, to realize improvement of impedance matching behavior. These EM absorbers, such as included Fe/graphene, FeCo/graphene, Fe_3_O_4_/Mexnes, and so on, showed the distinct enhancement performance as compared to the single components [16,17]. 

Expecting for components, absorber with various morphologies also would make a great influence on the performance. E.g., Xu et al., taking graphene hybrid as a case, observed that loading same component with different nanostructure (nanoparticle/nanosheet), the dielectric loss ability was changed [18]. They explained that the changed dielectric loss ability was caused by interfacial polarization effect, which was highly associated with nanostructure. On the basis of this finding, Cao et al. designed a multiple-interface hybrid (namely, *Co_3_O_4_@rGO/SiO_2_*) and achieved a *f_s_* value of 4.2 GHz (*covering the entire X band*) [19]. Similarly, Zhang et al. reported a Fe_3_Si/SiC@SiO_2_ absorber and got a *f_s_* value of 5.4 GHz (*d*~2.4 mm) [20]. All these desirable results confirmed the contribution of interfacial polarization. 

Inspired by these results, we prepared a core–shell structured EM absorber, using Co/Co_3_O_4_ as the cores. The magnetic loss behavior can be easily tuned by adjusting the content of Co. Initial, Co_3_O_4_ nanospheres are made by a hydrothermal route and then used as the source of magnetic Co. Afterward, the as-obtained Co_3_O_4_ was coated by amorphous carbon to induce carbon reduction. The temperature for carbon reduction played a key role on the final content of Co. The developed Co/Co_3_O_4_@C presented excellent wideband EM absorption performance. 

## 2. Experimental

### 2.1. Preparation of Co_3_O_4_ Nanospheres

Co_3_O_4_ nanospheres were prepared by a hydrothermal and annealing process. Typically, 0.1 g PVP, Co(Ac)_2_, and urea (~20 mg) were codissolved in a mixture solvent, containing EG (20 mL) and distilled water (20 mL) and maintain at pH = 13. The above solution was used for the hydrothermal reaction, which was heated at 140 °C for 6 h. Once cooled to room temperature, the precipitate was collected by centrifugation for two to three times with distilled water. Later, Co_3_O_4_ was obtained by directly heating the precipitate at 300 °C for 0.5 h. Argon gas (Ar) was used as the protective gas.

### 2.2. Synthesis of Co/Co_3_O_4_@C Hybrids

Typically, 0.1 g Co(OH)_2_ nanosphere, 0.4 g phenolic resin, and 0.2 mL formaldehyde were added into solution (~60 mL distilled water) and stirred for 2 h. Then, the as-obtained precipitates were heated at 500, 600, and 700 °C under Ar atmosphere for 1 h with a slow ramping rate of 1 °C/min. The obtained products treated at 500, 600, and 700 °C were denoted as C-Co-500, C-Co-600, and C-Co-700, respectively.

### 2.3. Characterization

The composition and phase of samples were determined by an X-ray diffractometer (Bruker D8 ADVANCE X-ray diffractometer) in the range of 20–60°. Field emission scanning electron microscope (FESEM, JEOL JSM-6330F) and transmission electron microscope (TEM, JEOL 2100) were used to observe the morphologies of these specimens. The chemical valences of Co element were evaluated by an X-ray photoelectron spectroscopy (XPS, PHI 5000 VersaProbe systems). Raman spectrum (Jobin Yvon HR 800 confocal Raman system) was employed to detect the graphitization level of the amorphous carbon shell. The coercive force (*Hc*) and magnetization were recorded by a vibrating sample magnetometer (VSM, Lakeshore, model 7400 series) at room temperature. EM characteristics were evaluated on basis of a coaxial-line theory. First, the composites used for the EM absorption measurement were prepared by mixing the CoxOy@C with paraffin wax in 50 wt%. Afterwards, a cylindrical-shaped sample (*Φ*_in_ = 3.04 mm, *Φ*_out_ = 7.0 mm) was made by hot pressing the mixture into a mold. The electromagnetic parameters were tested by the two-port vector network analyzer (Agilent E5071C). Finally, the reflection loss value was calculated based on following equations [21,22,23]:

*Z_in_* = *Z_o_*(*μ_r_*/*ε_r_*)^1/2^tanh[*j*(2*πfd*(*μ_r_ε_r_*)^1/2^/*c*)] (1)

*RL*(*dB*) = 20log|(*Z_in_* − *Z_o_*)/(*Z_in_* + *Z_o_*)| (2)

where *Z_in_* is the input impedance of absorber, *f* relates to the frequency of electromagnetic wave, *d* represents the coating thickness of the absorber, while *c* is the light velocity. *ε_r_* (*ε_r_* = *ε*′ − *jε*″) and *µ_r_* (*µ_r_* = *µ*′ − *jµ*″) are the complex permittivity and permeability of the absorption layer. 

## 3. Results and Discussion

**Figure 1:**
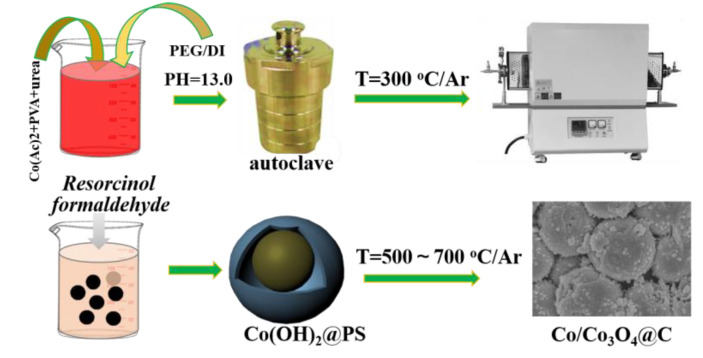
Schematic presentation for the preparation route of Co/Co_3_O_4_@C composites.

The formation process of Co/Co_3_O_4_@C spherical-shaped hybrids are illustrated in Figure 1. First, the spherical-shaped Co(OH)_2_ samples are fabricated by a hydrothermal route. Second, Co(OH)_2_ is converted to Co_3_O_4_ via annealing products at 300 °C for 1 h (Figure S1). The diffraction peaks intensity of Co_3_O_4_ and Co(OH)_2_ are very strong, which are due to the good crystalline structure. Co(OH)_2_@ phenolic resin (PS: the precursor of amorphous carbon) is made by an in situ polymerization route. By heating the Co(OH)_2_@PS at various temperatures (here is 500–700 °C), Co/Co_3_O_4_@C with various content of Co can be obtained. Figure S2 compares the EDS and FTIR spectra of Co(OH)2@PS and C-Co-500 sample. The EDS spectra of Co(OH)_2_@PS and Co/Co_3_O_4_@C samples are provided in Figure S2a,b, where the C, Co, and O elements can be detected on both samples. Hence, it is hard to observe the changes of component. Subsequently, the FT-IR spectra have been added to compare the changes of chemical bonds (Figure S2c,d). Clearly, the wave number at around 575 cm^−1^ is assigned to the characteristic vibrating peak of Co(OH)_2_ and is consistent with the XRD result. After heating at 500 °C, the C-OH peak disappeared and turned to two types of Co-O bonds, known as CoO_4_ (550 cm^−1^) and CoO_6_ (670 cm^−1^), suggesting the coexisted spinel phase of Co_3_O_4_. Additionally, due to the carbonized reaction, the original C-H bond disappeared for the C-Co-500 sample. 

**Figure 2:**
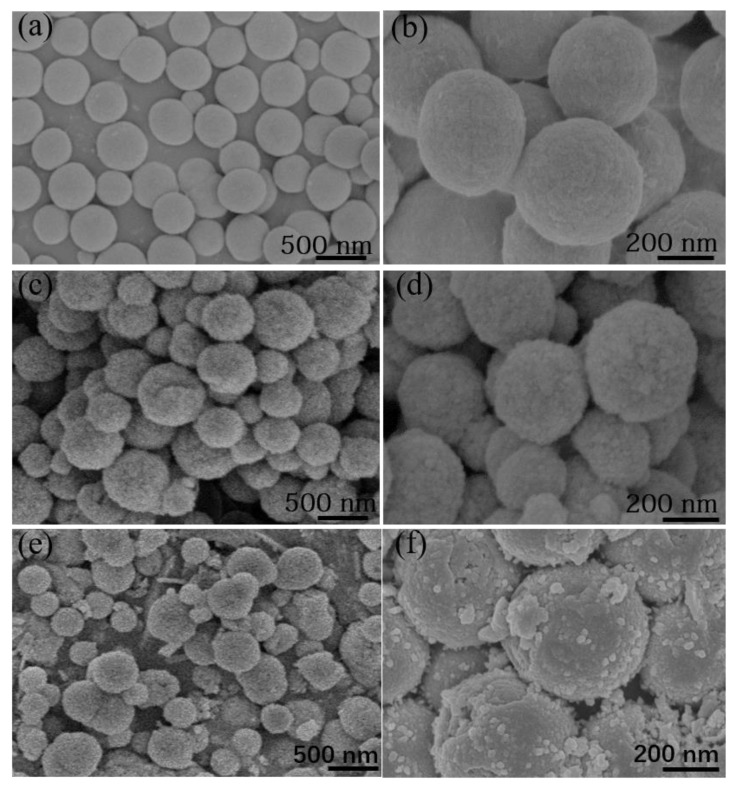
FESEM images of the products as received at different stages: (**a**,**b**) Co(OH)_2_, (**c**,**d**) Co_3_O_4_, and (**e**,**f**) the representative Co/Co_3_O_4_@C as treated at 700 °C.

Figure 2 shows the morphology evolution from initial Co(OH)_2_ to ultimate Co/Co_3_O_4_@C. Co(OH)_2_ exhibits significant spherical-shaped structure with average size of ~400 nm (Figure 2a,b). The surface of Co(OH)_2_ is very smooth and dense. As for Co_3_O_4_, it still maintains original spherical shape with the same size, according to Figure 2c,d. But Co_3_O_4_ exhibits a tiny rough surface attributing to the loss of phase conversion from Co(OH)_2_ to Co_3_O_4_. Similarly, structure can be maintained for Co/Co_3_O_4_@C (C-Co-700), expecting for the surface (Figure 2e,f) to be rough, which was attributed to the amorphous carbon shell. 

**Figure 3:**
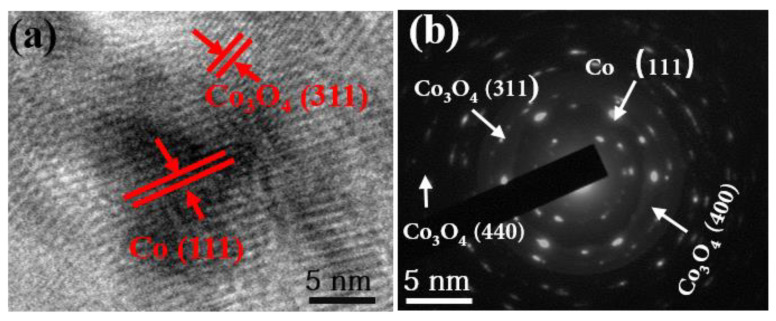
(**a**) HRTEM and (**b**) SAED image of C-Co-700 product.

Figure 3 shows the HRTEM images of C-Co-700 sample. The lattice with the distance of 0.24 nm corresponds to the (111) crystal plane of the Co phase. The lattice with the distance of 0.21 nm can be ascribed to the (311) plane of Co_3_O_4_. It shows one selected area of electron diffraction pattern, which is indexed to the (311), (440), and (400) planes of Co_3_O_4_ and (111) crystal plane of Co, respectively. 

**Figure 4:**
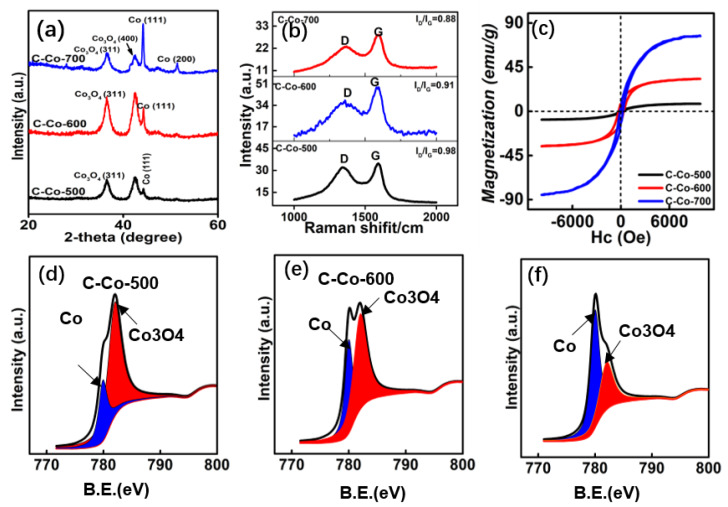
(**a**) XRD patterns, (**b**) Raman spectrum, (**c**,**d**) *M-H* curves of Co/Co_3_O_4_@C samples treated at various temperatures, and (**e**,**f**) XPS spectrums for the Co 3/2 p.

The crystal structures for Co/Co_3_O_4_@C are characterized by X-ray pattern (XRD). As shown in Figure 4a, the diffraction peaks at 36.2° and 42.7° are assigned to the (311) and (400) crystal planes of Co_3_O_4_. Besides, other diffraction peaks at 44.2° and 51.1° can be ascribed to the (111) and (200) crystal planes of Co. Furthermore, the intensity of Co signals is gradually stronger as it increases for the product treated with a high temperature, revealing the increased content of Co. As for amorphous carbon shell, there is no obvious signal, because of amorphous state. To confirm existence of carbon, the Raman spectrums are employed here, as plotted in Figure 4b. Clearly, two noticeable peaks at 1360 and 1590 cm^−1^, representing D and G band, respectively, are found for these Co/Co_3_O_4_@C hybrids [24]. It is widely accepted that G band is generated by the graphitized carbon atom which adopts sp^2^ hybridized form [25]. D band is induced by the crystal defects or disorders existing in carbon materials [26]. Commonly, the ratio of D/G band is an indicator of the graphitization degree. In our case, the ratios are estimated to be 0.98, 0.91, and 0.88 for the C-Co-500, C-Co-600, and C-Co-700, respectively. The increased graphitization degree is ascribed to a high carbonization temperature, according to recent achievement [27]. The magnetization loops (M-H) curves were measured by a vibrating sample magnetometer (VSM) (Figure 4c,d). In general, the magnetization value has a vital correlation with permeability parameters, includes real part of permeability and magnetic loss value (*μ*′/*μ*″), as can be expressed by the following equations [28]:

*μ*′ = 1 + (*M*/*H*)*cosσ* (3)

*μ*″ = (*M*/*H*) *sinσ* (4)

where *M* represents the magnetization, *H* means the external magnetic field, and σ refers to the phase lag angle of magnetization behind external magnetic field. From Equations (3) and (4), a high magnetization value is related to a bigger *μ*′ and *μ*″ values, thus benefiting to the impedance matching and magnetic loss ability. In Figure 4c, the magnetization values are estimated to be 9.0, 33, and 75 emus/g for C-Co-500, C-Co-600, and C-Co-700 samples. The increased magnetization value confirms the enhancement of Co content [29]. The X-ray photoelectron spectrum (XPS) is also performed to analyze the valances of Co. The binding energy values of Co 2p_3/2_ are located at 782.1 and 778.2 eV, which can be corresponded to Co_3_O_4_ and Co, respectively (Figure 4e–g) [30]. The surface area ratio of S_Co/SCo_3_O_4__ are 0.23, 0.43, and 0.81 for C-Co-500, C-Co-600, and C-Co-700, respectively, representing the molar ratio. 

**Figure 5:**
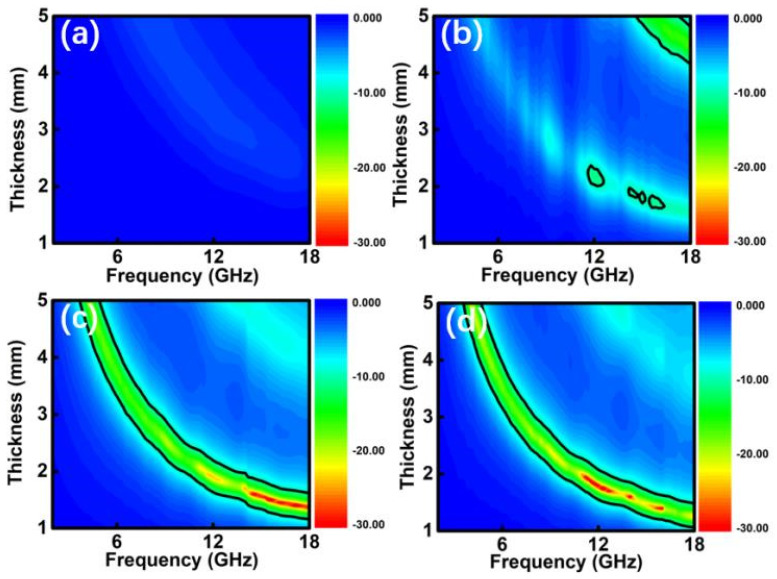
Color-mappings of reflection loss curves for the Co/Co_3_O_4_@C and Co_3_O_4_ samples: (**a**) Co_3_O_4_, (**b**) C-Co-500, (**c**) C-Co-600, and (**d**) C-Co-700.

**Figure 6:**
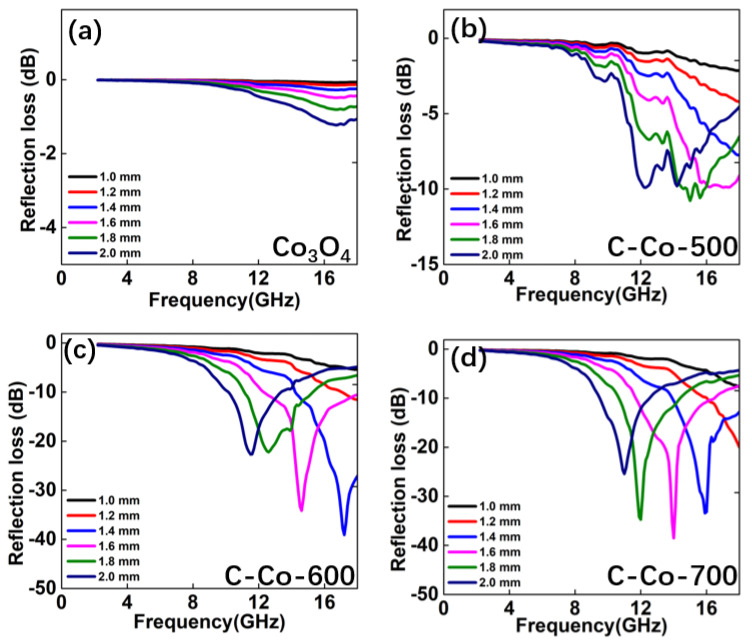
Frequency dependent of reflection loss curves for the Co/Co_3_O_4_@C and Co_3_O_4_ samples: (**a**) Co_3_O_4_, (**b**) C-Co-500, (**c**) C-Co-600, and (**d**) C-Co-700; noted that the thickness < 2.0 mm.

Figure 5 plots the frequency dependence of two-dimensional reflection loss (RL) curves. Considering the transmission line theory, the reflection loss values are calculated using the measured data of relative permittivity and permeability at a given frequency region (2–18 GHz) and thickness layer (1–5 mm). Obviously, the absorption layer filled with Co_3_O_4_ exhibits the poor EM performance, due to the unqualified RL values (<−10 dB) [31]. But significant improvement can be found for these Co/Co_3_O_4_@C sample. In fact, a desirable EM absorber is requested to a broadband absorption for the thickness < 2.0 mm. To clarify it, the reflection loss curves at 1.0–2.0 mm are given in Figure 6. In such a thickness region, *RL_min_* values of Co_3_O_4_ are all higher than −2.0 dB, thus cannot be used as absorber. For C-Co-500 product, the minimum *RL_min_* value of −10.8 dB is achieved under a thickness of 1.8 mm. In other thickness, there is no frequency region with RL < −10 dB. As the content of Co is increased, the absorption intensity becomes distinctly stronger. The minimum *RL_min_* value can up to −39.4 dB for C-Co-600, while the thickness in only 1.4 mm (Figure 6c). Meanwhile, the qualified frequency region covers 4.3 GHz, ranging from 13.8 to 18.0 GHz. While for C-Co-700 (Figure 6d), the lowest *RL_min_* value can be gained is −38.6 dB under a matched thickness of 1.6 mm. The corresponding *f_s_* value is 4.9 GHz. For comparison, the EM performance of Co or C containing hybrids are listed in Table 1, demonstrating that the Co/Co_3_O_4_@C hybrids shows improvement of EM absorption ability [32,33,34,35,36,37,38,39].

**Figure 7:**
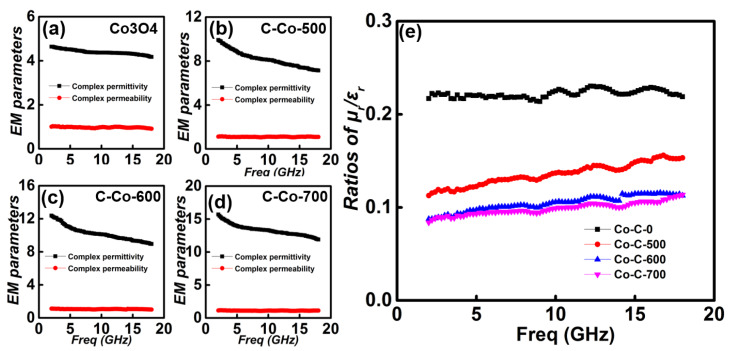
Frequency dependence of *μ_r_*/*ε_r_* curves for the Co/Co_3_O_4_@C and Co_3_O_4_ samples: (**a**) Co_3_O_4_, (**b**) C-Co-500, (**c**) C-Co-600, (**d**) C-Co-700, and (**e**) the ratios of *μ_r_*/*ε_r_* for the Co/Co_3_O_4_@C and Co_3_O_4_ sample.

It should be noted that EM absorption performance is always determined by the impedance matching and EM attenuation ability. The key factor for the impedance matching ability can be estimated by the ratio of complex permeability/permittivity (*μ_r_*/*ε_r_*) [40]. Figure 7 plots the curves of *μ_r_*, *ε_r_*, and their ratios of *μ_r_*/*ε_r_*. Due to the nonmagnetic characteristic, *μ_r_* of Co_3_O_4_ is almost a constant of 1.0, according to Figure 7a. *ε_r_* decreases from 4.6 to 4.2. As compared to Co_3_O_4_, *μ_r_* of C-Co-500 is only a little higher than Co_3_O_4_, attributing to the low content of Co (Figure 7b). But significant enhancement can be found for *ε_r_* ranging in 7.2~9.9. By further increasing the content of Co, both *μ_r_* and *ε_r_* increase, e.g., *μ_r_* values are ~1.1 and 1.2 for C-Co-600 and C-Co-700, respectively (Figure 7c,d). Meanwhile, *ε_r_* of C-Co-700 is highest and distributed in the region 15.9~12. The ratios of *μ_r_*/*ε_r_* values are then applied to estimate the impedance matching ability (Figure 7e). The ratio of Co_3_O_4_ is distinct larger than these Co/Co_3_O_4_@C samples, revealing the good impedance matching behavior. In this case, it can be deduced that the poor EM performance of Co_3_O_4_ is primarily due to the weaken attenuation ability. For these Co/Co_3_O_4_@C hybrids, the ratios of C-Co-600 is much closer to C-Co-700, and all are smaller than C-Co-500.

**Figure 8:**
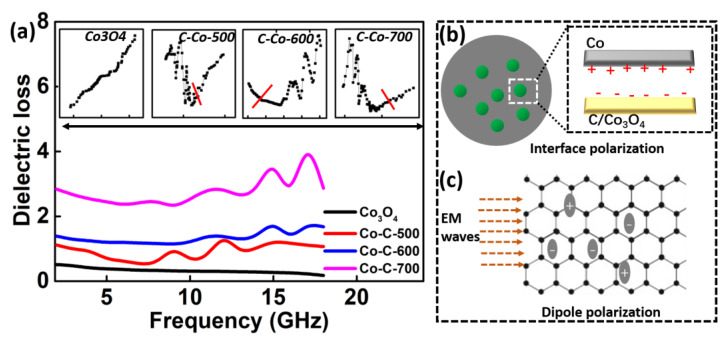
Frequency dependence of dielectric loss curves for the Co/Co_3_O_4_@C and Co_3_O_4_ samples: the schematic illustration of proposed interface (**b**,**c**) dipole polarization mechanism; noted the inserted images in Figure 8 (**a**) represents the Cole–Cole curves.

Commonly, the mechanism for EM attenuation results from dielectric and magnetic loss. Figure 8a plots the *ε*″-*f* curves for these products. It can be clearly seen that Co_3_O_4_ achieves the lowest *ε*″ value (~0.35), representing the worst dielectric loss ability. For Co/Co_3_O_4_@C samples, the *ε*″ slowly decreases first as the frequency increases. Then, multiple dielectric loss peaks are observed in high-frequency region (*f* > 6.5 GHz), revealing polarization relaxation behavior. In such a frequency region (*f* > GHz), polarization forms primarily included are interfacial and dipole polarization, according to recent discussions on mechanism [41,42]. In general, either dipole or interfacial polarization relaxation effect can be revealed by the Cole–Cole semicircle. In detail, the relative complex permittivity can be described by the following equations [43,44,45,46]:

(5) $$\varepsilon_{r}=\varepsilon_{\infty }+\frac{\varepsilon_{s}-\varepsilon_{\infty }}{1+j2\pi f\tau }=\varepsilon^{\prime }-j\varepsilon^{\prime \prime }$$

where *ε_s_*, *ε*_∞_, and *τ* are static permittivity, relative dielectric permittivity at high-frequency limit, and polarization relaxation time, respectively, whereas, *ε*′ and *ε*″ can be calculated based on the following equations.

(6) $$\varepsilon^{\prime }=\varepsilon_{\infty }+\frac{\varepsilon_{s}-\varepsilon_{\infty }}{1+{\left(2\pi f\right)}^{2}\tau^{2}}$$

(7) $$\varepsilon^{\prime \prime }=\frac{2\pi f\tau (\varepsilon_{s}-\varepsilon_{\infty })}{1+{\left(2\pi f\right)}^{2}\tau^{2}}$$

Based on Equations (6) and (7), the *ε*′-*ε*″ can be expressed as above:

(8) $${\left(\varepsilon^{\prime }-\varepsilon_{\infty }\right)}^{2}+{\left(\varepsilon^{\prime \prime }\right)}^{2}={\left(\varepsilon_{s}-\varepsilon_{\infty }\right)}^{2}$$

If the plot of *ε*′-*ε*″ is a semicircle, it represents one Debye polarization relaxation process which make a contribution for *ε*″. Commonly, such a semicircle is termed as Cole–Cole semicircle. The inserted images in Figure 8a show the Cole–Cole curves of these products. It can be revealed that only Co/Co_3_O_4_@C products exhibit Cole–Cole semicircles, indicating the polarization behavior. The proposed mechanisms for polarization are illustrated in Figure 8b,c. In our case, the developed Co/Co_3_O_4_@C exhibits multiple interfaces, including Co/Co_3_O_4_, Co/C, and Co_3_O_4_/C. When an external EM field is provided, the electrons from the Co will be attracted by Co_3_O_4_ or groups of amorphous carbon, because of difference in electronegativity. Consequently, the interfacial relaxation process occurs, favoring the dielectric loss. In addition to interfacial polarization, dipole polarization may also attribute to the dielectric loss value. 

Amorphous carbon shell always contains various forms of defects, such as crystal defects, presence of C-containing groups, e.g., –COOH, –C=O, –COH, etc. (Figure 8c). These defects can act as the dipole center and induce dipole polarization. Such a dipole process has a contribution for ε″ [47]. 

**Figure 9:**
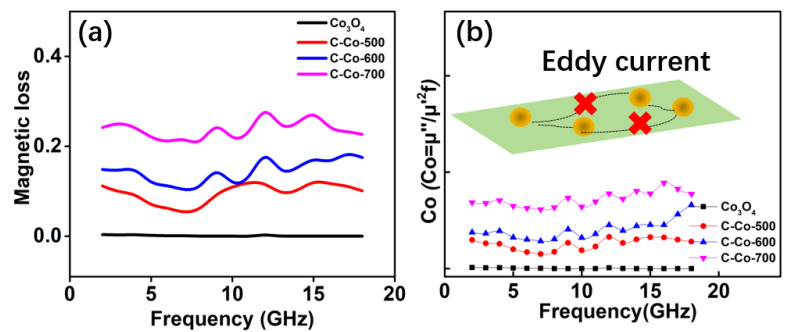
(**a**) Frequency dependence of magnetic loss curves for the Co/Co_3_O_4_@C and Co_3_O_4_ samples and (**b**) the Co-f curves of the four products.

Magnetic loss ability is discussed in Figure 9. Because of the biggest content of Co, C-Co-700 has a largest *μ*″ value, equaling to 0.24. Meanwhile, *μ*″ versus frequency exhibits multiple peaks. As we know, *μ*″ mainly comes from hysteresis loss, domain wall resonance, natural resonance, exchange resonance, and eddy current effect [45]. The hysteresis loss arising from irreversible magnetization is negligible in a weakly applied field, and the domain wall resonance occurs only in multidomain materials and usually in 1–100 MHz region, and thus, the contributions from hysteresis loss and domain wall resonance to magnetic loss can be excluded in our material system. If the magnetic loss originates from eddy current effect, the values of $C_{0}(C_{0}=\mu^{\prime \prime }{\left(\mu^{\prime }\right)}^{-2}f^{-1}=2\pi \mu_{0}\sigma d^{2}/3)$ should be a constant when the frequency increases [48,49]. The *C*_0_ values of all samples fluctuate at full frequency region (Figure 9b). Hence, we can deduce that the magnetic loss mainly results from natural resonance and exchange resonances. 

The attenuation constant *α* represents the integral loss ability, including magnetic or dielectric loss, which can be calculated by following equation [50]:

(9) $$\alpha =\frac{\sqrt{2}\pi f}{c}\times \sqrt{\left(\mu^{\prime \prime }\varepsilon^{\prime \prime }-\mu^{\prime }\varepsilon^{\prime }\right)+\sqrt{{(\mu^{\prime \prime }\varepsilon^{\prime \prime }-\mu^{\prime }\varepsilon^{\prime })}^{2}+(\mu^{\prime }\varepsilon^{\prime \prime }+\mu^{\prime \prime }\varepsilon^{\prime }{)}^{2}}}$$

**Figure 10:**
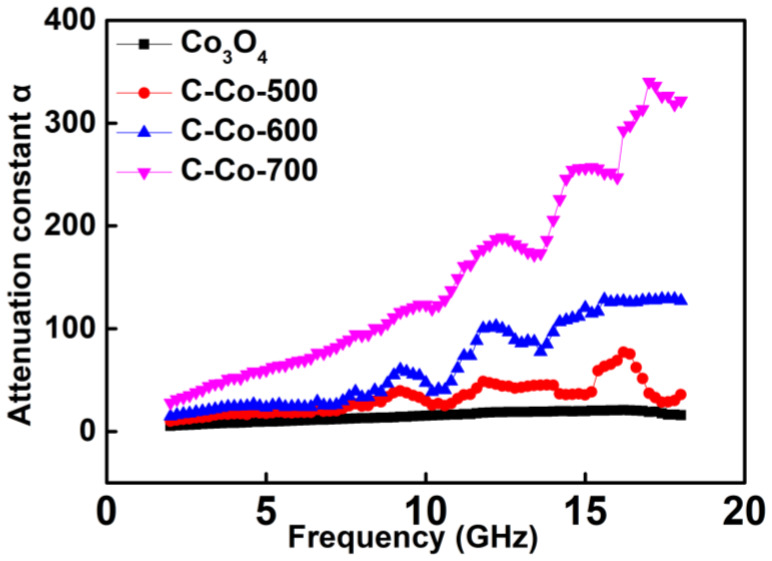
Wave-attenuation constant α for these products.

From Figure 8 and Figure 9, we can find that C-Co-700 sample has the highest dielectric and magnetic losses, thus leading to a biggest attenuation constant α (Figure 10). The content of Co plays a key role on tuning the dielectric and magnetic loss ability. It also has proven that Co_3_O_4_ has a quite lower attenuation constant α. Overall, C-Co-600 achieves the best EM performance which attributes to the several factors. First, the Co/Co_3_O_4_ as the core has efficiently prevented the eddy current effect. Second, the suitable component of Co ensures the strong interfacial polarization, which is beneficial to dielectric loss. In addition, a suitable content of Co also maintains a moderately ε_r_ value, hence enabling to balance the impedance matching ability. 

## 4. Conclusions

In this work, a Co/Co_3_O_4_@C product was developed by a facile hydrothermal and annealing method. First, the spherical-shaped Co_3_O_4_ with an average size of ~400 nm was prepared and then coated by carbon. The as-obtained Co_3_O_4_@C was heated at various temperatures, resulting in Co/Co_3_O_4_@C hybrids. The content of Co was tunable by only controlling the temperatures. The EM performance were studied, based on the transmission line theory. The minimum reflection loss value (RL_min_) of −39.4 dB could be achieved for the Co/Co_3_O_4_@C sample. The corresponding thickness was only 1.4 mm. Furthermore, the frequency region with *RL* < −10 dB, was up to 4.3 GHz, covering 13.7~18.0 GHz. The excellent electromagnetic absorption mechanism was discussed in depth, which was attributed to the multi-interface-induced interface polarization. Meanwhile, the existed magnetic Co enabled balancing the impedance matching behavior. 

## Figures and Tables

**Table 1:** Electromagnetic (EM) performances of Co- or C-based hybrids as reported by recent literatures.

Samples	RL_min_ (dB)	Effective Absorption Region (GHz)	Thickness (mm)	Ref.
Co@C	~−10	<3.0	1.5	[32]
Porous carbon	−23.8	~3.8	2.0	[33]
CoO@Co/ZnO/graphene	−51.5	4.7	2.6	[34]
CoNi@C	−24.03	4.32	2.5	[35]
Co/ZnO/C	−52.6	4.9	3.0	[36]
Co_3_O_4_/graphene	−31.88	3.4	2.0	[37]
Co_3_O_4_@PANI	−37.39	~4.2	4.0	[38]
Co/CoO	−50 dB	4.2	2.0	[39]
C-Co-600	−39.4 dB	4.3	1.4 mm	This work

## Supplementary Materials

The following are available online at https://www.mdpi.com/2079-4991/10/5/902/s1, Figure S1: XRD patterns of Co(OH)_2_ and Co_3_O_4_, Figure S2: (a,b) EDS and (c,d) FR-IR spectral of Co(OH)_2_@PS and C-Co-500.
