# Supplementary material for: An Easy Method of Synthesis CoxOy@C Composite with Enhanced Microwave Absorption Performance
Source: Nanomaterials (Basel). 2020 May 8;10(5):902. doi: 10.3390/nano10050902 (PMC7279402; doi:10.3390/nano10050902)
Supplement: Supplementary file 1 [file nanomaterials-10-00902-s001.pdf]

An easy method of synthesis Co_xO_y@C composite with enhanced microwave absorption performance

Wenli Bao [a, 1], [*] Cong Chen [a,b, 1], Zhenjun Si[a,*]

[a] *School of Materials Science and Engineering, Changchun University of Science and Technology, No. 7989, Weixing Road, Changchun, 130022, PR China*

[b]*School of Physics and Electronic Information Engineering, Qinghai Nationalities University, Xining 810007, PR China*

[1] The first two authors contributed equally to this paper.

---

*Corresponding authors. *E-mail addresses*: zdbwl@163.com (W. Bao)

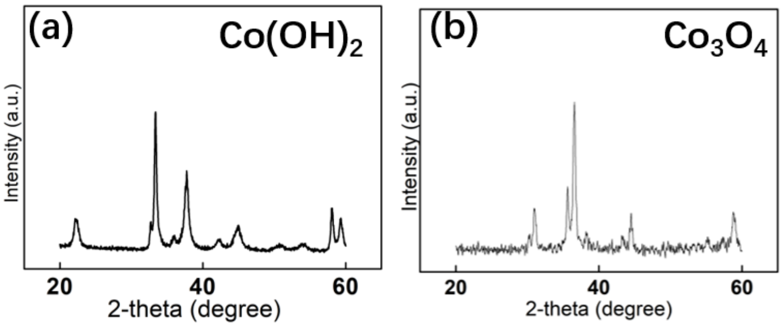

Figure S1 XRD patterns of $Co(OH)_2$ and Co_3O_4

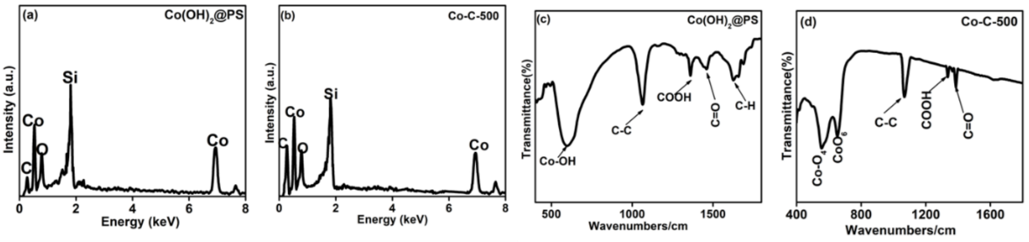

Figure S2: (a-b) EDS and (c-d) FR-IR spectral of $Co(OH)_2$@PS and C-Co-500
